# Identification and Validation of Immune-Related Long Non-Coding RNA Signature for Predicting Immunotherapeutic Response and Prognosis in NSCLC Patients Treated With Immunotherapy

**DOI:** 10.3389/fonc.2022.899925

**Published:** 2022-07-04

**Authors:** Jianli Ma, Minghui Zhang, Jinming Yu

**Affiliations:** ^1^ Department of Radiotherapy, Shandong University Cancer Center, Jinan, China; ^2^ Department of Medical Oncology, Harbin Medical University Cancer Hospital, Harbin, China

**Keywords:** non-small cell lung cancer, lncRNAs, immunotherapy, prognosis, lncRNA signature

## Abstract

**Background:**

Numerous studies have reported that long non-coding RNAs (lncRNAs) play important roles in immune-related pathways in cancer. However, immune-related lncRNAs and their roles in predicting immunotherapeutic response and prognosis of non-small cell lung cancer (NSCLC) patients treated with immunotherapy remain largely unexplored.

**Methods:**

Transcriptomic data from NSCLC patients were used to identify novel lncRNAs by a custom pipeline. ImmuCellAI was utilized to calculate the infiltration score of immune cells. The marker genes of immunotherapeutic response-related (ITR)-immune cells were used to identify immune-related (IR)-lncRNAs. A co-expression network was constructed to determine their functions. LASSO and multivariate Cox analyses were performed on the training set to construct an immunotherapeutic response and immune-related (ITIR)-lncRNA signature for predicting the immunotherapeutic response and prognosis of NSCLC. Four independent datasets involving NSCLC and melanoma patients were used to validate the ITIR-lncRNA signature.

**Results:**

In total, 7,693 novel lncRNAs were identified for NSCLC. By comparing responders with non-responders, 154 ITR-lncRNAs were identified. Based on the correlation between the marker genes of ITR-immune cells and lncRNAs, 39 ITIR-lncRNAs were identified. A co-expression network was constructed and the potential functions of 38 ITIR-lncRNAs were annotated, most of which were related to immune/inflammatory-related pathways. Single-cell RNA-seq analysis was performed to confirm the functional prediction results of an ITIR-lncRNA, LINC01272. Four-ITIR-lncRNA signature was identified and verified for predicting the immunotherapeutic response and prognosis of NSCLC. Compared with non-responders, responders had a lower risk score in both NSCLC datasets (P<0.05). NSCLC patients in the high-risk group had significantly shorter PFS/OS time than those in the low-risk group in the training and testing sets (P<0.05). The AUC value was 1 of responsiveness in the training set. In melanoma validation datasets, patients in the high-risk group also had significantly shorter OS/PFS time than those in the low-risk group (P<0.05). The ITIR-lncRNA signature was an independent prognostic factor (P<0.001).

**Conclusion:**

Thousands of novel lncRNAs in NSCLC were identified and characterized. In total, 39 ITIR-lncRNAs were identified, 38 of which were functionally annotated. Four ITIR-lncRNAs were identified as a novel ITIR-lncRNA signature for predicting the immunotherapeutic response and prognosis in NSCLC patients treated with immunotherapy.

## Introduction

According to the latest GLOBOCAN 2020 data, lung cancer is the second most commonly diagnosed malignancy with an estimated 2.2 million new cases (11.4%), and is the leading cause of cancer-related death accounting for 1.8 million (18% of the total cancer deaths) worldwide, with its number of new cases just behind female breast cancer (1). Non-small cell lung cancer (NSCLC) is the major histological type, and accounts for approximately 80-85% of all lung cancers (2, 3). While surgery, chemotherapy, radiation therapy, and targeted therapy are commonly used in the clinical treatment for NSCLC patients, there were certain limitations. For instance, patients treated with targeted therapy inevitably develop drug resistance (3–5). Recently, immunotherapy has been widely used to treat patients with NSCLC. Immune checkpoint blockade has dramatically changed the prognosis of NSCLC patients (2, 6), whereas long-lasting benefits are only seen in a subgroup of patients (2, 7). Therefore, research on molecular biomarkers in responders is critical for predicting responsiveness and prognosis.

Most studies on clinical biomarkers have focused on protein-coding genes, while few have focused on long non-coding RNAs (lncRNAs), which are defined as non-coding RNAs longer than 200 nucleotides in length with low or no protein-coding potential. Previous studies have explored the functions of lncRNAs, and found that they participate in many biological processes, such as cell proliferation, apoptosis, immune response, cancer immunity, and immune system (8–14). Immune-related pathways play crucial roles in tumor development and progression. In addition, increasing studies have reported that immune-related lncRNA signature could be used to predict the prognosis of various cancer types, including breast cancer, bladder cancer, NSCLC, renal clear cell cancer, and hepatocellular carcinoma (15–19). However, few studies have focused on pre-immunotherapy transcriptomic profiles to predict the immunotherapeutic response and prognosis of NSCLC patients.

Based on the pre-immunotherapy transcriptomic data of NSCLC, we aimed to systematically identify and characterize novel lncRNAs for NSCLC, assess tumor microenvironments, identify and annotate immune-related lncRNAs, and construct a prognostic signature for predicting the immunotherapeutic response and prognosis of NSCLC patients treated with immunotherapy.

## Materials and Methods

### Datasets Collection, Reads Mapping, and Transcripts Assembly

In this study, pre-immunotherapy transcriptomic profiles, survival information, and annotation information of cell clusters were downloaded from the Gene Expression Omnibus (GEO, http://www.ncbi.nlm.nih.gov/geo) and the European Nucleotide Archive (ENA, https://www.ebi.ac.uk/ena/browser/home), including 2 bulk RNA sequencing (bulk-RNA-seq) datasets and one single-cell RNA sequencing (scRNA-seq) dataset from NSCLC patients, and three bulk-RNA-seq datasets from melanoma patients (20–24). Raw bulk-RNA-seq data from NSCLC patients were used to identify novel lncRNAs, and identify and validate immunotherapeutic-response-immune-related (ITIR)-lncRNAs prognostic signature. ScRNA-seq data from NSCLC was used to validate the potential functions of ITIR-lncRNA. In order to validate the reliability of the risk model in another cancer type, three melanoma datasets were used as independent testing sets, which included patients treated with anti-PD-1 monotherapy or combined with ipilimumab immunotherapy.

Raw bulk-RNA-seq data was analyzed by FastQC v0.11.3 (http://www.bioinformatics.babraham.ac.uk/projects/fastqc/) for quality statistics summary. Adapters and low-quality sequences were removed by TrimGalore-0.6.0 (https://www.bioinformatics.babraham.ac.uk/projects/trim_galore/) with default parameters. Clean reads were aligned to the human reference genome (version hg38/GRCh38) by STAR v.2.7.8a (25, 26) with the *twopassMode* set as Basic. The bam files of each patient were *de novo* assembled by StringTie v2.1.6 (27). Assembled transcripts from each patient were merged by the cuffmerge function (Cufflinks v2.2.1) (28). Kallisto v.0.46.2 (29) was used to calculate the reads counts and transcripts per million (TPM) value with default parameters.

### Identification of Novel lncRNAs in NSCLC Patients

To identify novel lncRNAs in NSCLC patients, firstly, the cuffcompare function of Cufflinks package (28) was used to compare the difference between primary assembled transcripts with human reference genome from GENCODE v38 (30) and RefLncRNA (31) genes annotation, respectively. According to the “class code” information outputted by the cuffcompare function, the merged assembled transcripts were classified into four categories, including completely matched (=), partially matched (j), contained (c), and not matched. Based on the potential novel lncRNAs catalog derived from NSCLC patients, a custom pipeline (32) was used to identify the reliable novel lncRNAs by the following criteria: a. the class codes are “i, x, u”; b. transcript lengths >= 200 nt and exon numbers >= 2; c. non-coding sequences reported by both CPC2 (Coding Potential Calculator) (33) and CNCI (Coding Noncoding Index) (34); d. recurrence >= 2.

### Identification of ITR-lncRNAs and ITR-mRNAs in NSCLC

Not appreciably expressed genes were removed, which were expressed in less than two samples, and the sum of count values<10. The R “DESeq2” package was used to calculate immunotherapeutic response-related (ITR)-lncRNAs and ITR-mRNAs by comparing responders with non-responders in two NSCLC datasets, respectively. P value<0.05 and | log2 fold change (log2FC) | >1 served as the cutoff criteria. The intersection analysis was performed of ITR-lncRNAs and ITR-mRNAs in two NSCLC datasets, respectively.

### Identification of IR-lncRNAs and ITIR-lncRNAs in NSCLC

The ESTIMATE algorithm (R “estimate” package) was utilized to calculate the immune score in each patient to assess the overall immune status. Riaz’s algorithm (35) was used to calculate the score of immune-related signatures in each patient. The ImmuCellAI algorithm (36) was performed to calculate the infiltration score of 24 types of tumor-infiltrating immune cells in each patient to investigate the tumor microenvironments. The one-tailed Wilcoxon test was used to compare the difference in immune status, immune-related signature, and tumor microenvironments between responders and non-responders. Based on the specific marker genes of ITR-immune cells, immune-related (IR)-lncRNAs were identified by Pearson correlation analysis (R “psych” package) with the cutoff criteria (P<0.05 and r^2^>0.7). Through the intersection analysis, ITIR-lncRNAs were identified.

### Construction of Co-Expression Network

Pearson correlation analysis (R “psych” package) was used to calculate the correlation between ITIR-lncRNA and mRNA. The lncRNA-mRNA pairs were selected with the cutoff criteria (adjust P value<0.05, r^2^>0.55 and ranked in the top 100). mRNA-mRNA pairs were selected with the cutoff criteria (adjust P value<0.05 and r^2^>0.8). Based on the lncRNA-mRNA pairs and mRNA-mRNA pairs, a co-expression network was constructed. The co-expression network was produced by Cytoscape 3.8.2 (37).

### scRNA-Seq Data Processing

Based on scRNA-seq data, we profiled the transcriptomes of ~45000 cells from 11 early-stage NSCLC samples. Cells and genes filtering were performed as follows: cells without annotation information were removed. Genes with low expression levels (nfeature<200) and expressed in less than three cells were removed. The R “Seurat” package was used to normalize and hierarchical clustering the cells by the standard procedures in each patient, respectively. The “TSNEPlot” and “Vlnplot” method was used to visualize the cell clustering and/or expression levels of CD68, CD163, and LINC01272 in all cell clusters in each NSCLC patient.

### Construction and Validation of ITIR-lncRNAs Prognostic Signature

In the training set, LASSO regression analysis (R “glmnet” package) and multivariate Cox regression analysis (R “survival” and “survminer” packages) were used to screen prognosis-related ITIR-lncRNAs and construct the risk model. The risk score for each patient was calculated based on the expression levels (log2-transformed TPM value) of ITIR-lncRNAs, and was calculated by the following formula:


$$Risk\;score=\sum_{n=1}^{n}\left(Coef_{i}\times Expression\;level_{ITIR-lncRNA\;i}\right)$$

According to the third quantile value of risk score, NSCLC patients were divided into the high-risk and low-risk groups. Kaplan-Meier (K-M) curves analysis (R “survival” and “survminer” packages) and receiver-operating characteristic (ROC) (R “pROC” package) were used to evaluate the clinical prognostic capacity of the risk score.

Four independent datasets, including one NSCLC dataset and three melanoma datasets, were used to validate the ITIR-lncRNA signature. The risk score formula was performed to calculate the risk score of each patient. In each testing set, patients were divided into high-risk and low-risk groups according to the same cutoff as the training set. The survival analysis and ROC analysis were performed as well.

Moreover, univariate and multivariate Cox regression analyses were used to evaluate whether ITIR-lncRNA signature can be regarded as an independent predictor of prognosis of NSCLC patients among other clinical information, including age and gender.

### Gene Functional Enrichment Analysis

Functional and pathway enrichment analyses were performed using the online database “Metascape” (38) website (http://metascape.org).

### Statistical Analysis

All statistical analyses were conducted using the R software version 4.1.1 (https://www.r-project.org/). Forest plots were plotted using the R “forestplot” package. Other packages in R were used in the study including “ggplot2”, “ggpubr” and “pheatmap”. The significance level was set at 0.05 (P<= 0.05).

## Results

### Construction of Novel lncRNA Catalog for NSCLC Patients Under Immunotherapy

**Figure 1:**
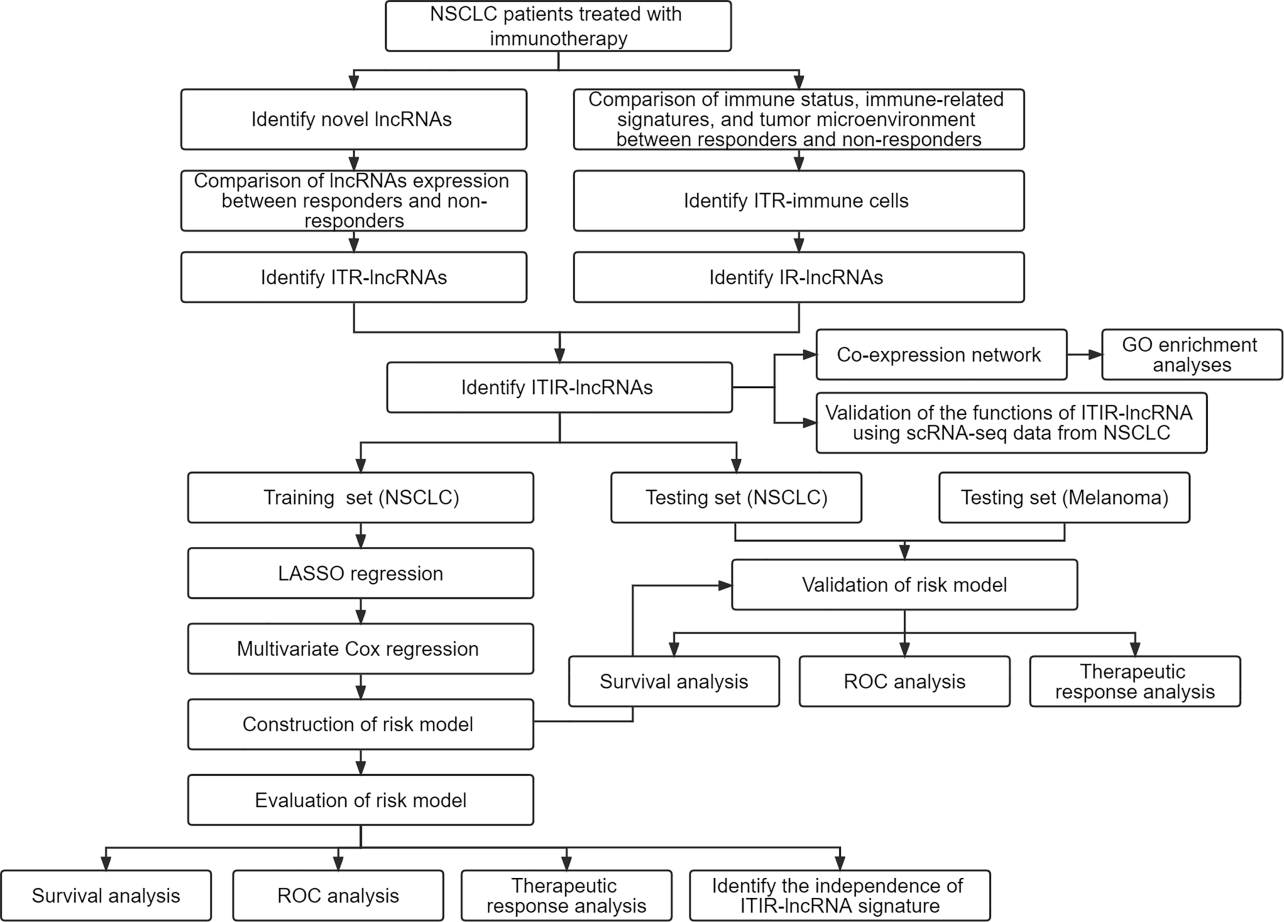
The study design and overall workflow. NSCLC, non-small cell lung cancer; ROC, receiver-operating characteristic; lncRNAs, long non-coding RNAs; ITR, immunotherapeutic response-related; IR, immune-related; ITIR, immunotherapeutic-response-immune-related.

**Figure 2:**
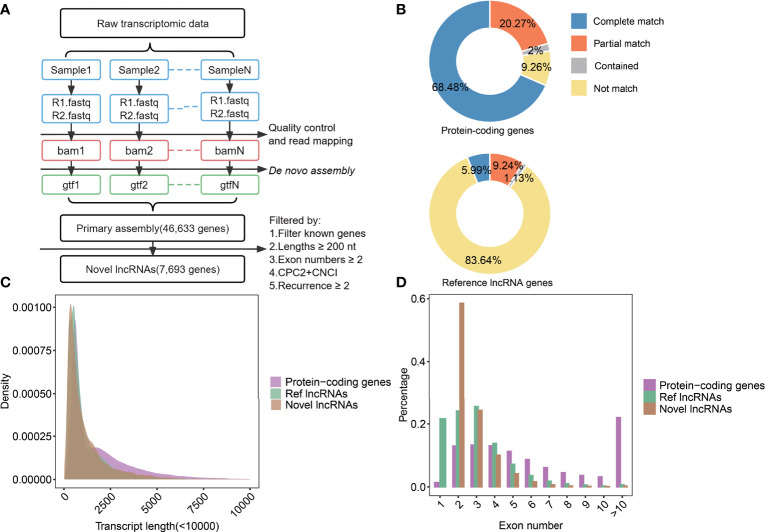
The identification process of novel lncRNAs and analysis in their characters. **(A)** The scheme of pipelines was used to identify novel lncRNAs. **(B)** The statistics of assembled transcripts matched to protein-coding genes (top) and reference lncRNA genes (bottom). **(C)** Density diagrams showed the transcript lengths in protein-coding genes, Ref lncRNAs, and novel lncRNAs. **(D)** Bar plot showed exon numbers in protein-coding genes, Ref lncRNAs, and novel lncRNAs.

To explore immune-related lncRNAs and their potential roles in NSCLC patients under immunotherapy, raw bulk-RNA-seq data from NSCLC were used to identify novel lncRNAs (study design shown in **Figure 1**). Through *de novo* assembly and transcripts merging, a total of 46,633 primary genes were identified (**Figure 2A** and **Supplementary Figures 1A–D**). By comparing with the reference genes annotation, we found that 90.74% (18,118/19,966) of protein-coding genes could be verified, and 68.48% (13,672/19,966) were completely matched (**Figure 2B**). In contrast, 16.36% (9,735/59,489) of known lncRNAs could be verified, and 5.99% (3,566/59,489) were completely matched (**Figure 2B**). Subsequently, the primary constructed transcripts that did not match with the reference genes annotation were used for the following analyses. In total, 7,693 novel lncRNAs were identified (32). Furthermore, we analyzed the transcript lengths and exon numbers of novel lncRNAs. The results showed that the distribution of transcript lengths (mean=1.2k nt) and exon numbers (93% were ranged from 2 to 4) of novel lncRNAs were close to ReflncRNAs (**Figures 2C, D**).

### Identification of ITR-lncRNAs

Based on the lncRNA profile, we systematically analyzed ITR-lncRNAs in NSCLC patients treated with immunotherapy. By comparing responders with non-responders, 154 ITR-lncRNAs (including 44 novel lncRNAs, **Supplementary Figures 2A–C** and **Supplementary Table 1**) and 251 ITR-mRNAs (**Supplementary Figures 3A–D** and **Supplementary Table 2**) were identified. To further explore the functions of these genes, GO enrichment analysis was performed and found that up-regulated genes were enriched in immune-related pathways, including T cell activation, myeloid leukocyte activation, and positive regulation of immune response, which were consistent with the previous study (35)(**Supplementary Figure 3E** and **Supplementary Table 3**). Notably, these pathways are frequently involved in the modulation of the immune environment (39). These findings suggested that ITR-lncRNAs may affect the efficacy of immunotherapy by influencing immune response-associated pathways.

### Tumor Microenvironment Analysis and Identification of ITIR-lncRNAs

**Figure 3:**
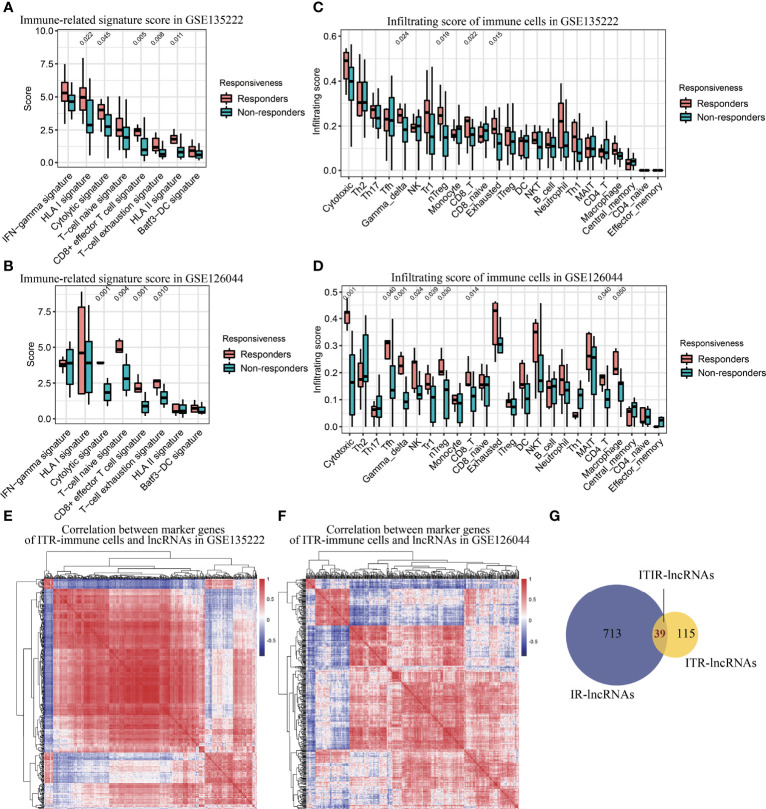
Immune infiltration analysis and identification of ITIR-lncRNAs. **(A)** Boxplot showed the score of immune-related signatures in responders and non-responders in the GSE135222 dataset. **(B)** Boxplot showed the score of immune-related signatures in responders and non-responders in the GSE126044 dataset. **(C)** Boxplot showed the infiltration score of 24 types of immune cells in responders and non-responders in the GSE135222 dataset. **(D)** Boxplot showed the infiltration score of 24 types of immune cells in responders and non-responders in the GSE126044 dataset. **(E)** Heatmap showed the correlation between marker genes of ITR-immune cells and IR-lncRNAs in the GSE135222 dataset. **(F)** Heatmap showed the correlation between marker genes of ITR-immune cells and IR-lncRNAs in the GSE126044 dataset. **(G)** Venn diagram showed the overlapped lncRNAs between ITR-lncRNAs and IR-lncRNAs.

To further investigate immune regulation-related (IR)-lncRNAs in NSCLC, we compared the immune status, immune-related signature, and immune cells infiltration scores between responders and non-responders. Compared with non-responders, responders had a significantly higher immune score in both NSCLC datasets (P<0.05, **Supplementary Figure 4**). Responders had significantly higher scores of cytolytic, HLA-I, HLA-II, T-cell naïve, T-cell exhaustion, and CD8+ effector T cell signature than non-responders (P<0.05, **Figures 3A, B**). In addition, responders had significantly higher immune cells infiltration scores, including cytotoxic T cells, Tfh cells, γδ T cells, NK cells, Tr1 cells, nTreg cells, CD8 T cells, exhausted T cells, CD4 T cells, and macrophages than non-responders (P<=0.05, **Figures 3C, D**). Based on the specific marker genes of ITR-immune cells (**Table 1**), 752 IR-lncRNAs were identified by the correlation analysis (**Figures 3E, F**). Through the intersection analysis of the ITR-lncRNAs and IR-lncRNAs, 39 ITIR-lncRNAs were obtained (**Figure 3G**).

**Table 1:** Specific marker genes of ITR-immune cells.

Cell type	Marker gene
CD8+ T	CD8A, CD8B
CD4+ T	CD4
Cytotoxic T	GNLY, GZMA, GZMB, GZMH, GZMK
γδT	IFNG, KLRD1, KLRK1
nTreg	FOXP3, CTLA4
Exhausted T	LAG3, TIGIT, PDCD1, TOX
Tfh	CCR5, TNFSF4, CD40LG
Macrophage	CD68, CD163
NK	NCAM1, NKG7

### Investigation of the Functions of ITIR-lncRNAs by Co-Expression Network Analysis

**Figure 4:**
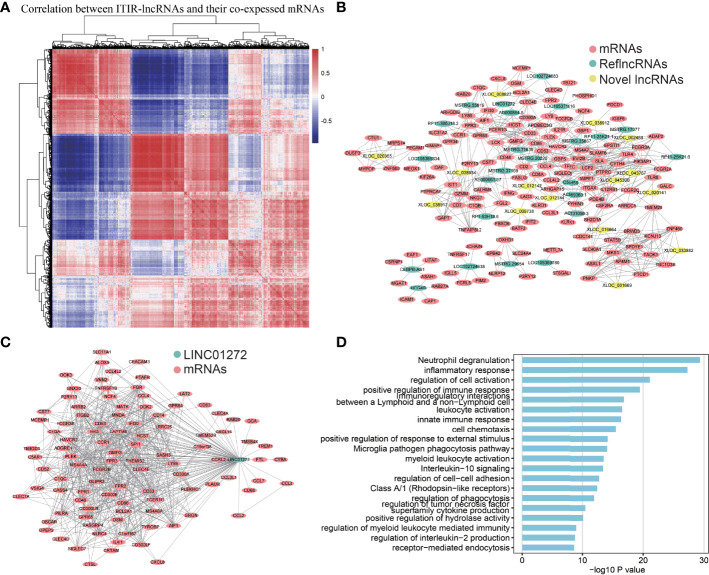
Co-expression network and functional annotation of ITIR-lncRNAs. **(A)** Heatmap showed the correlation between 39 ITIR-lncRNAs and their top100 highly co-expressed mRNAs in NSCLC. **(B)** The co-expression network showed the relationship between 39 ITIR-lncRNAs and their top5 highly co-expressed mRNAs in NSCLC. Colored by different types of RNAs. **(C)** The co-expression network showed the relationship between LINC01272 and its top100 highly co-expressed mRNAs in NSCLC. Colored by different types of RNAs. **(D)** Barplots showed the top 20 GO enrichment pathways of the LINC01272 (P < 0.05).

To further explore the functions of the 39 ITIR-lncRNAs, a co-expression network was constructed. Based on the correlation between 39 ITIR-lncRNAs and mRNAs, 3,503 lncRNA-mRNA pairs were identified, including 39 ITIR-lncRNAs and 1,299 mRNAs (**Figures 4A, B** and **Supplementary Table 4**). GO enrichment analysis revealed that the protein-coding genes in the co-expression network were mainly enriched in immune-related pathways, including leukocyte activation, regulation of cell activation, positive regulation of cytokine production, inflammatory response, innate immune response, and so on (**Supplementary Figure 5** and **Supplementary Table 5**). Accordingly, the 39 ITIR-lncRNAs involved in the co-expression network may play similar roles with their co-expressed coding genes.

**Figure 5:**
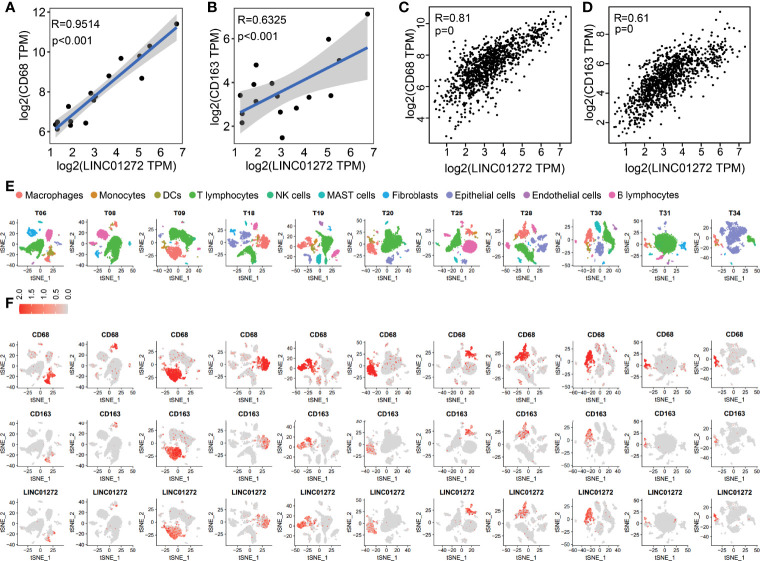
Validation of the functions of LINC01272 using scRNA-seq data in NSCLC. **(A)** The scatter plot showed the correlation between LINC01272 and CD68 in NSCLC dataset from the GEO database. **(B)** The scatter plot showed the correlation between LINC01272 and CD163 in NSCLC dataset from the GEO database. **(C)** The scatter plot showed the correlation between LINC01272 and CD68 in NSCLC dataset from the GEPIA database. **(D)** The scatter plot showed the correlation between LINC01272 and CD163 in NSCLC dataset from the GEPIA database. **(E)** The tSNE projection within each patient was colored by ten cell types, including macrophages, monocytes, DCs, T lymphocytes, NK cells, MAST cells, fibroblasts, epithelial cells, endothelial cells, and B lymphocytes. **(F)** The tSNE plot showed expression levels of CD68 (top), CD163 (middle), and LINC01272 (bottom) in each NSCLC patient.

Furthermore, we performed GO enrichment analysis for each ITIR-lncRNA. The functions of the 38 ITIR-lncRNAs were successfully annotated (**Supplementary Table 6**). The annotation results showed that 33 ITIR-lncRNAs were related to immune regulation and immune response, and the other ITIR-lncRNAs were related to Wnt signaling or cell cycle-related pathways. Notably, an ITIR-lncRNA named LINC01272, which was mainly involved in “inflammatory response”, “immune response”, and “regulation of phagocytosis” (**Figures 4C, D**), was positively correlated with CD68 and CD163 (**Figures 5A, B**), which act as the specific markers of macrophages. This result was validated using a larger dataset in the GEPIA database (**Figures 5C, D**). To further validate the potential functions of LINC01272 in macrophages, we performed deep analyses using scRNA-seq data involving 44,900 cells from NSCLC. As shown in **Figure 5E**, cells in each patient were classified into ten clusters, including macrophages, monocytes, DCs, T lymphocytes, NK cells, MAST cells, fibroblasts, epithelial cells, endothelial cells, and B lymphocytes. Macrophages, monocytes, and DCs were characterized by high expression of CD68, and were further distinguished by the specific expression of CD163 (**Figure 5F** and **Supplementary Figure 6**). LINC01272 was also specifically expressed in macrophages and monocytes, especially macrophage clusters (**Figure 5F** and **Supplementary Figure 6**), implying that it plays an important role in macrophages. The above findings suggested that ITIR-lncRNAs with immune regulation functions have great potential applications in immunotherapy prognosis and immune response-related markers.

### Construction and Evaluation of the ITIR-lncRNA Prognostic Signature

**Figure 6:**
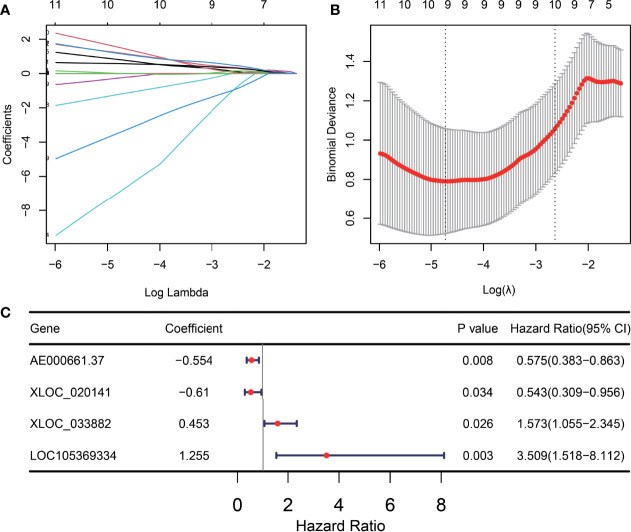
Construction of the ITIR-lncRNA signature. **(A)** The distribution plot of the LASSO coefficient. Nine variables were retained when Log Lambda was equal to –4.73. **(B)** Nine variables were retained when the partial likelihood deviation reached the minimum (Log Lambda = –4.73). **(C)** The Forest plot showed the coefficient, p-value, and hazard ratio (HR) of four ITIR-lncRNAs by using the multivariate Cox regression analysis.

Based on 39 ITIR-lncRNAs, we constructed a risk model for predicting the immunotherapeutic responses and prognosis of NSCLC patients treated with immunotherapy. LASSO regression analysis was used, and nine ITIR-lncRNAs were retained when log lambda was equal to –4.73 and the partial likelihood deviation reached the minimum (**Figures 6A, B**). Subsequently, multivariate Cox regression was used to screen for prognosis-related ITIR-lncRNAs, and four ITIR-lncRNAs were identified for modeling, including AE000661.37, XLOC_020141, XLOC_033882, and LOC105369334. (P<0.05, **Figure 6C**). The risk score was calculated for each patient.

**Figure 7:**
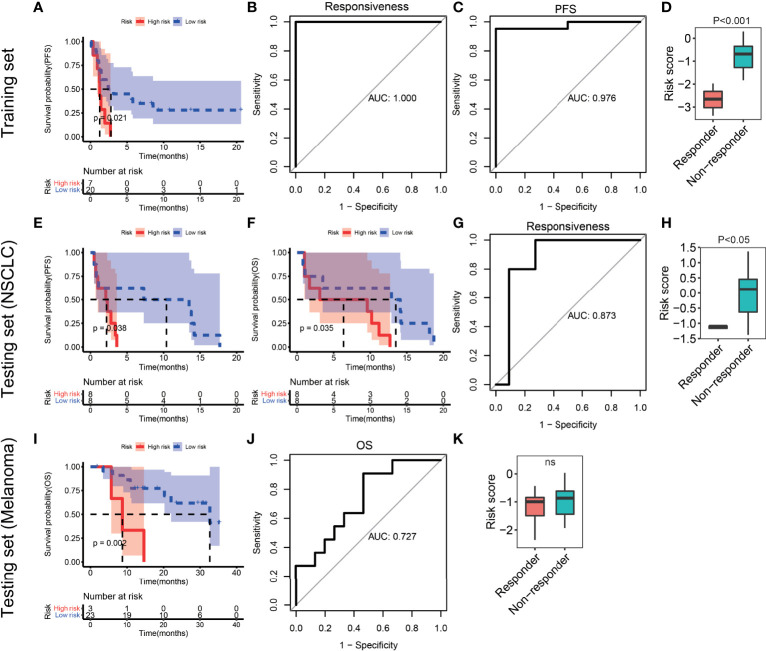
Evaluation and validation of the ITIR-lncRNA signature in NSCLC and melanoma datasets. **(A)** Kaplan-Meier analysis of PFS comparing the high-risk (red) group with the low-risk group (blue) in the training set. **(B)** ROC curves for responsiveness in the training set. **(C)** ROC curves for PFS in the training set. **(D)** Boxplot of risk score comparing responders with non-responders in the training set. **(E, F)** Kaplan-Meier analysis of PFS and OS comparing the high-risk (red) group with the low-risk group (blue) in the NSCLC testing set. **(G)** ROC curves for responsiveness in the NSCLC testing set. **(H)** Boxplot of risk score comparing responders with non-responders in the testing NSCLC dataset. **(I)** Kaplan-Meier analysis of OS comparing the high-risk (red) group with the low-risk group (blue) in the melanoma dataset. **(J)** ROC curves for OS in the melanoma dataset. **(K)** Boxplot of risk score comparing responders with non-responders in the melanoma dataset.

According to the third quantile value of the risk score in the training set, NSCLC patients were classified into the high-risk and low-risk groups. Patients in the high-risk group had significantly shorter progression-free survival (PFS) time than those in the low-risk group (P=0.021, **Figure 7A**). The area under the curve (AUC) of the ITIR-lncRNA signature was 1 of responsiveness and 0.976 of PFS (**Figures 7B, C**). Compared with non-responders, responders had a significantly lower risk score (P<0.001, **Figure 7D**).

### Validation of the ITIR-lncRNA Prognostic Signature

To validate the reliability of the ITIR-lncRNA prognostic signature, four independent datasets were used, including one NSCLC dataset and three melanoma datasets. In the NSCLC dataset, patients were classified into the high-risk and low-risk groups according to the same cutoff of the risk score as the training set. Patients in the high-risk group had shorter PFS (P=0.038) and overall survival (OS, P=0.035, **Figures 7E, F**) than those in the low-risk group. The AUC was 0.873 of responsiveness (**Figure 7G**). Compared to non-responders, responders had a significantly lower risk score (P<0.001, **Figure 7H**).

In three melanoma datasets, the same methods were used. Patients treated with the anti-PD-1 monotherapy or combined with ipilimumab immunotherapy in the high-risk group had shorter survival period than those in the low-risk group (P<0.05, **Figure 7I** and **Supplementary Figures 7A, B**). The AUC values were 0.727, 0.662, and 0.648 of survival period in three melanoma dataset, respectively (**Figure 7J** and **Supplementary Figures 7C, D**). Additionally, we observed that responders had significantly lower risk scores than non-responders in two of three melanoma datasets (**Figure 7K** and **Supplementary Figures 7E, F**). The AUC values were 0.687 and 0.684 of responsiveness in two of three melanoma datasets, respectively (P<0.05, **Supplementary Figures 7G, H**).

### ITIR-lncRNA Signature Was an Independent Prognostic Factor

**Figure 8:**
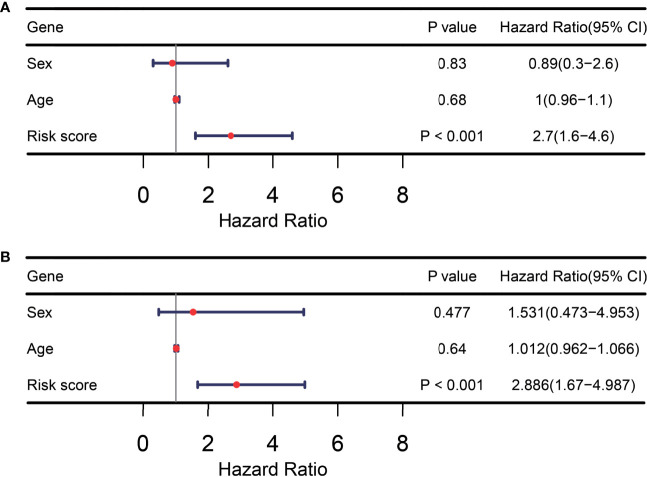
TIR-lncRNA signature was an independent prognostic factor for NSCLC patients. **(A)** The forest plot showed the results of univariate Cox regression analysis in the training set. **(B)** The forest plot showed the results of multivariate Cox regression analysis in the training set.

In addition, we assessed whether the ITIR-lncRNA signature was an independent prognostic factor for NSCLC among other clinical information, including age and gender. Univariate and multivariate Cox regression analyses revealed that the ITIR-lncRNA signature was an independent prognostic factor for NSCLC patients in the training set (P<0.001, **Figures 8A, B**).

## Discussion

In this study, based on transcriptome data from NSCLC patients treated with immunotherapy, we utilized systematic methods to identify novel lncRNAs and ITIR-lncRNAs for NSCLC, and constructed a prognostic signature for predicting the immunotherapeutic response and prognosis of NSCLC patients treated with immunotherapy. A total of 7,693 novel lncRNAs were identified and characterized in NSCLC based on raw transcriptomics data. By comparing responders with non-responders, ITR-lncRNAs and ITR-mRNAs were obtained. Next, we systematically investigated the differences in immune status, immune-related signatures, and tumor microenvironments between responders and non-responders. Based on the specific marker genes of ITR-immune cells, IR-lncRNAs were obtained by the Pearson correlation analysis. Furthermore, 39 ITIR-lncRNAs were identified through the intersection analysis, and functionally characterized by the co-expression network and GO enrichment analysis. In total, 38 ITIR-lncRNAs were annotated successfully. ScRNA-seq analysis revealed that LINC01272 might play an important role in macrophages in NSCLC. Four prognosis-related ITIR-lncRNAs were screened by LASSO and multivariate Cox regression analyses. In the training set, NSCLC patients were classified into high-risk and low-risk groups based on the third quantile value of risk scores and K-M curves showed that patients in the high-risk group had a shorter PFS than those in the low-risk group. The AUC values were 1 of responsiveness and 0.976 of PFS. Four independent datasets were used to validate the prognostic model, including NSCLC patients and melanoma patients. Patients were classified into the high-risk and low-risk groups according to the same cutoff as the training set, and observed that patients in the high-risk group had shorter survival period than those in the low-risk group in testing sets. Taken together, we identified and validated a four-ITIR-lncRNA signature for predicting the immunotherapeutic response and prognosis of NSCLC patients treated with immunotherapy. Nevertheless, experimental validation of ITIR-lncRNA signature is lacking, which needs to be further explored.

There were four ITIR-lncRNAs in the risk model, including AE000661.37, XLOC_020141, XLOC_033882, and LOC105369334. AE000661.37 and LOC105369334 are known lncRNAs, while the other two lncRNAs are novel. Except for the two novel lncRNAs, very little is known about the role of these two known lncRNAs in cancer and cancer immunity. To further investigate the functions of ITIR-lncRNAs, functional enrichment analyses were performed. AE000661.37 was mainly involved in “leukocyte activation”, “Natural killer cell mediated cytotoxicity”, “innate immune response”, and so on (**Supplementary Table 6**). The top5 co-expressed coding genes with AE000661.37 were FGL2, KLRD1, CALHM6, FASLG, and CST7. FGL2 is a member of the fibrinogen superfamily, which plays an immunosuppressive factor in the tumor microenvironment. Overexpression of FGL2 can predict worse survival in esophageal carcinoma (40). However, another study found that the expression level of FGL2 correlated with better prognostic outcomes of lung adenocarcinoma (41). LOC105369334 was mainly involved in “G beat gamma signaling through PI3Kgamma”, “Cell migration and invasion through p75NTR”, “Wnt signaling pathway”, and so on (**Supplementary Table 6**). The top5 co-expressed coding genes with LOC105369334 were MEOX1, GIMAP1, PECAM1, KIF26A, and OAF. Recent studies have reported that MEOX1 plays an important role in breast cancer, ovarian cancer, and lung cancer (42–45). Meanwhile, GIMAP1, as a member of some novel gene signatures, can predict prognosis in pancreatic cancer (46), endometrial cancer (47), and breast cancer (48). XLOC_020141 was mainly involved in “leukocyte activation”, “macrophage activation”, “positive regulation of interleukin-1 beta production”, and so on (**Supplementary Table 6**). The top5 co-expressed coding genes with XLOC_020141 were TLR6, GALC, FCGR2C, TMEM26, and ARRDC5. TLR6 (transmembrane protein) is a member of the Toll-like receptor (TLR) family which plays an important role in the adaptive immune response. A recent study reported that TLR6, as a member of a novel gene signature, can predicts the prognosis of lung adenocarcinoma (49). TMEM26 is also a transmembrane protein that may act as a tumor suppressor by impeding the acquisition of endocrine resistance in breast cancer (50). XLOC_033882 was mainly involved in “Regulation of RUNX1 Expression and Activity”, “hemopoiesis”, “Endocytosis”, and so on (**Supplementary Table 6**). The top5 co-expressed coding genes with XLOC_033882 were SPDYE1, KCNJ13, TBC1D3B, ZNF460, and TAOK1. ZNF460 is a member of the ZNFs family, and overexpression of ZNF460 can predict worse survival in colon cancer (51). The mechanism of the functions of these four ITIR-lncRNAs needs to be further explored.

## Data Availability Statement

Publicly available datasets were analyzed in this study. The datasets generated and/or analyzed during the current study are available in the GEO repository (https://www.ncbi.nlm.nih.gov/geo/query/acc.cgi?acc=GSE135222, https://www.ncbi.nlm.nih.gov/geo/query/acc.cgi?acc=GSE126044, https://www.ncbi.nlm.nih.gov/geo/query/acc.cgi?acc=GSE78220, https://www.ncbi.nlm.nih.gov/geo/query/acc.cgi?acc=GSE131907) and ENA repository (https://www.ebi.ac.uk/ena/browser/view/PRJEB23709?show=reads).

## Author Contributions

Data analysis, JM, MZ, and JY; JM, MZ, and JY wrote the manuscript; JY supervised and conceived the study. All authors contributed to the article and approved the submitted version.

## Funding

This study was supported by Natural Science Foundation of China (NO. 81902329).

## Conflict of Interest

The authors declare that the research was conducted in the absence of any commercial or financial relationships that could be construed as a potential conflict of interest.

The handling editor CL declared a shared parent affiliation with the author MZ at the time of review.

## Publisher’s Note

All claims expressed in this article are solely those of the authors and do not necessarily represent those of their affiliated organizations, or those of the publisher, the editors and the reviewers. Any product that may be evaluated in this article, or claim that may be made by its manufacturer, is not guaranteed or endorsed by the publisher.
