# Supplementary material for: Identification and Validation of Immune-Related Long Non-Coding RNA Signature for Predicting Immunotherapeutic Response and Prognosis in NSCLC Patients Treated With Immunotherapy
Source: Front Oncol. 2022 Jul 4;12:899925. doi: 10.3389/fonc.2022.899925 (PMC9289523; doi:10.3389/fonc.2022.899925)
Supplement: Supplementary file 1 [file DataSheet_1.pdf]

Supplementary Figure 1

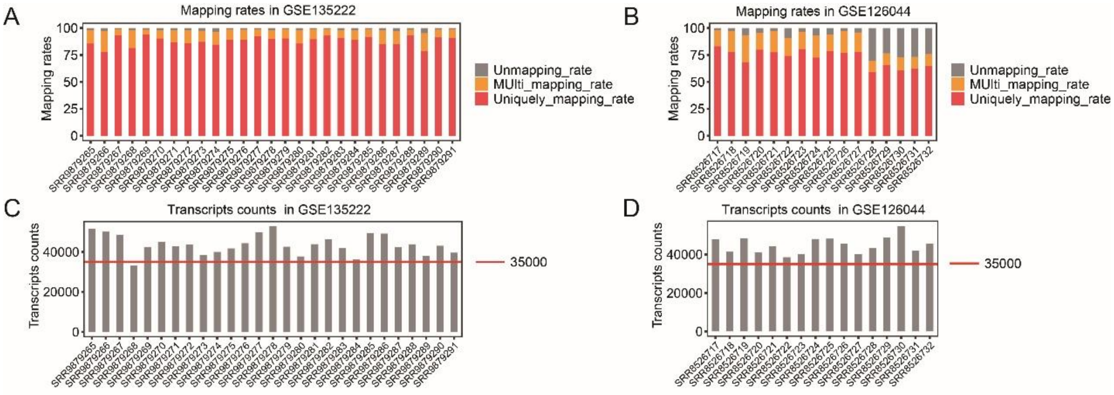

Supplementary Figure 1. Mapping rates and transcripts counts in two NSCLC datasets. (A, C) The bar plot showed the mapping rates in each patient in two NSCLC datasets, respectively. (B, D) The bar plot showed transcripts counts in each patient in two NSCLC datasets, respectively.

Supplementary Figure 2

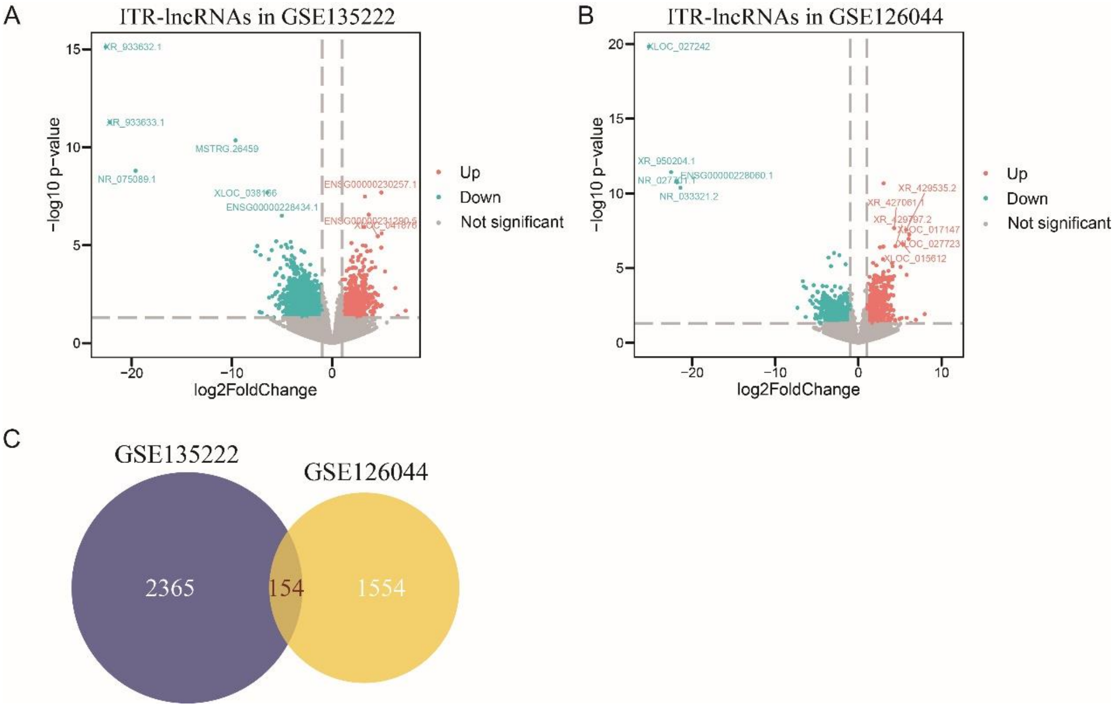

Supplementary Figure2. Identification of ITR-lncRNAs by comparing responders with non-responders in NSCLC. (A) The volcano plot showed ITR-lncRNAs in the GSE135222 dataset. (B) The volcano plot showed ITR-lncRNAs in the GSE126044 dataset. (C) Venn plot showed the overlapped ITR-lncRNAs between two NSCLC datasets.

Supplementary Figure 3

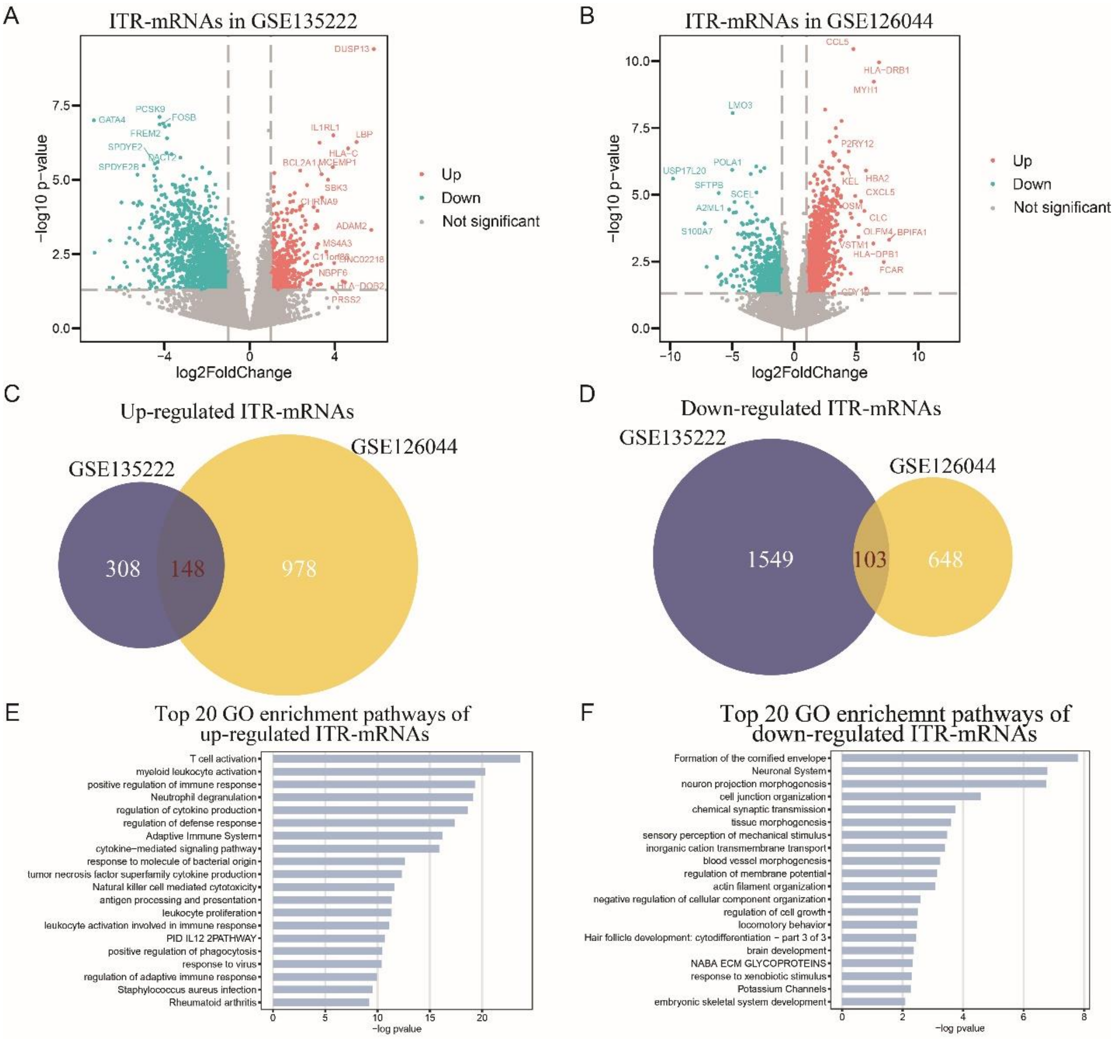

Supplementary Figure 3. Identification of ITR-mRNAs by comparing responders with non-responders in NSCLC. (A, B) The volcano plot showed ITR-mRNAs in the GSE135222 dataset. (B) The volcano plot showed ITR-mRNAs in the GSE126044 dataset. (C) Venn plot showed the overlapped up-regulated mRNAs between two NSCLC datasets. (D) Venn plot showed the overlapped down-regulated mRNAs between two NSCLC datasets. (E) The bar plot showed the top 20 GO enrichment pathways of the up-regulated genes. (F) The bar plot showed the top 20 GO enrichment pathways of the down-regulated genes.

Supplementary Figure 4

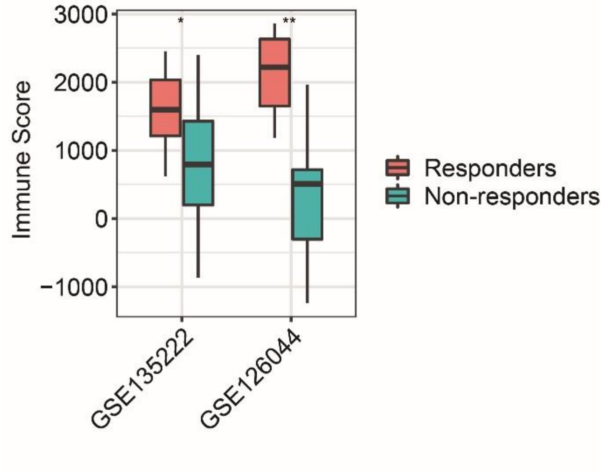

Supplementary Figure 4. Boxplot showed the immune score in responders and non-responders in two NSCLC datasets. * means p value < 0.05, ** means p value < 0.01, and *** means p value < 0.001.

Supplementary Figure 5

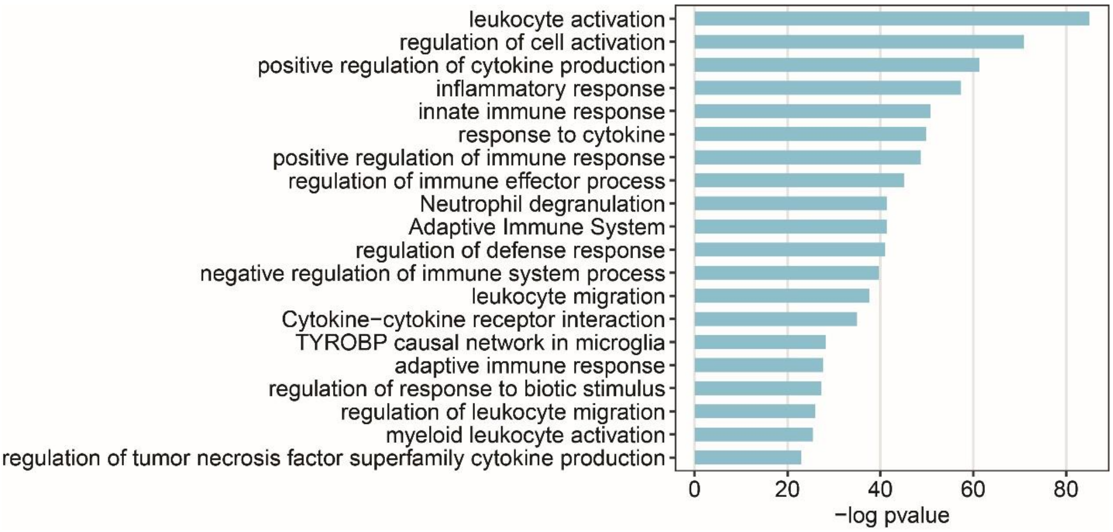

Supplementary Figure 5. The bar plot showed the top 20 GO enrichment pathways of the mRNAs in the co-expression network ($P < 0.05$).

Supplementary Figure 6

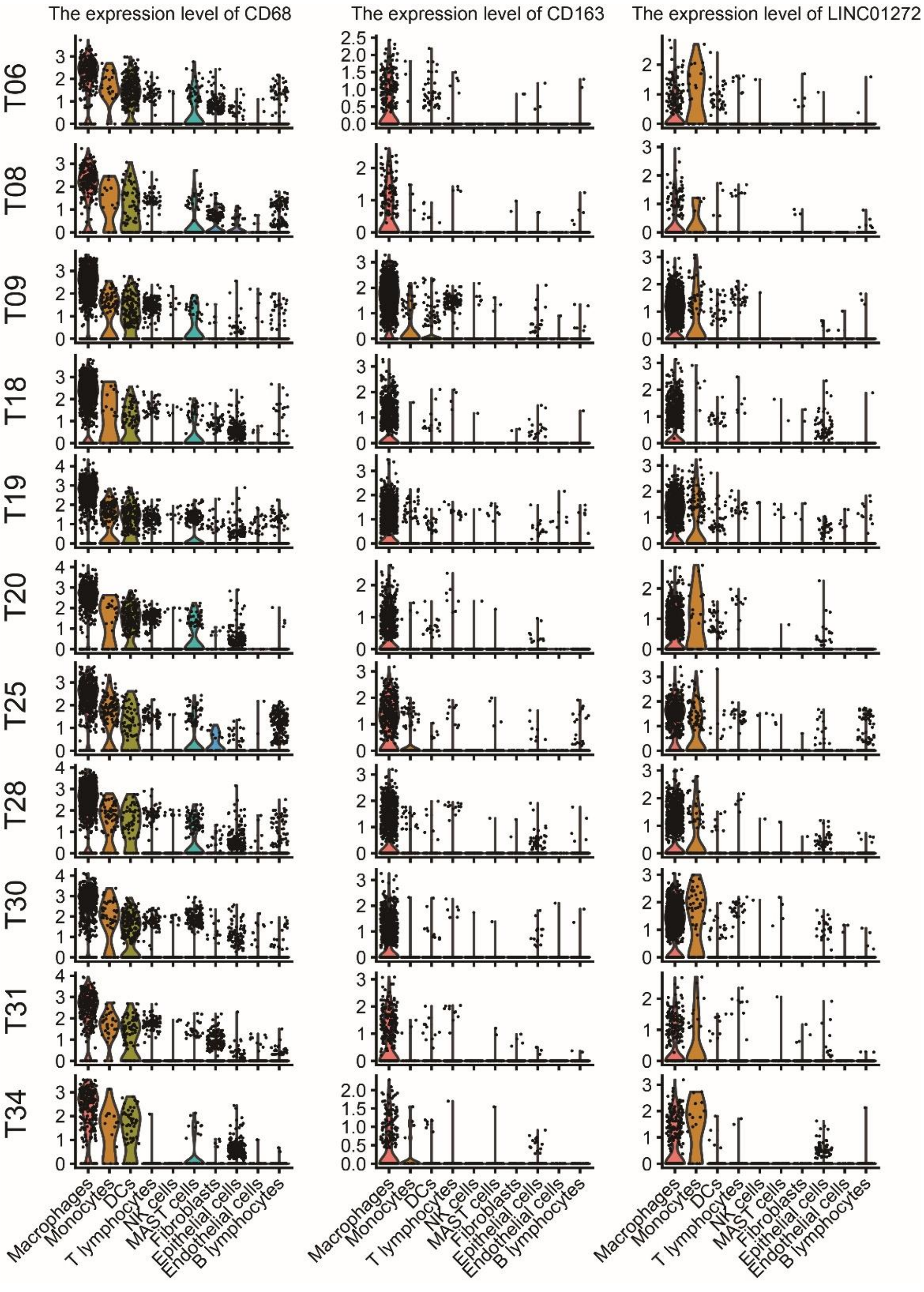

Supplementary Figure 6. Violin plot showed expression levels of CD68 (left), CD163 (middle), and LINC01272 (right) in all cell clusters in each NSCLC patient. Colored by different cell types.

Supplementary Figure 7

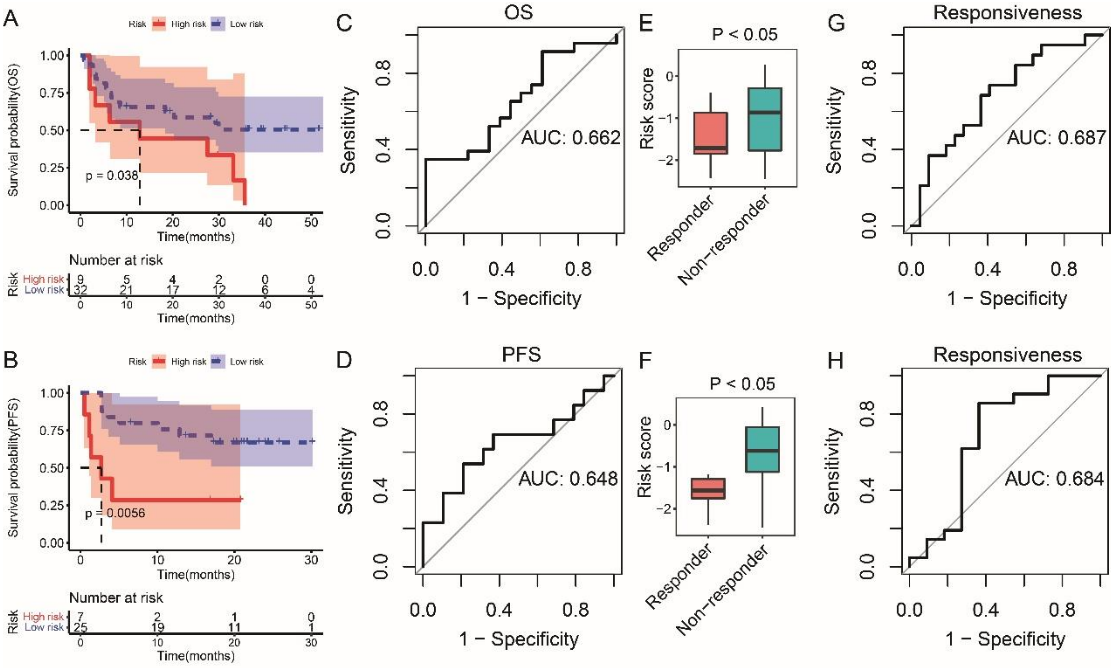

Supplementary Figure 7. Validation of the ITIR-lncRNA signature in additional two melanoma datasets. (A) Kaplan-Meier analysis of OS comparing the high-risk (red) group with the low-risk group (blue) in melanoma patients treated with anti-PD-1 monotherapy. (B) Kaplan-Meier analysis of PFS comparing the high-risk (red) group with the low-risk group (blue) in melanoma patients treated with combined ipilimumab and anti-PD-1 immunotherapy. (C) ROC curves for OS in the melanoma patients treated with anti-PD-1 monotherapy. (D) ROC curves for PFS in the melanoma patients treated with combined ipilimumab and anti-PD-1 immunotherapy. (E) Boxplot of risk score comparing responders with non-responders in the melanoma patients treated with anti-PD-1 monotherapy. (F) Boxplot of risk score comparing responders with non-responders in the melanoma patients treated with combined ipilimumab and anti-PD-1 immunotherapy. (G) ROC curves for responsiveness in the melanoma patients treated with anti-PD-1 monotherapy. (H) ROC curves for responsiveness in the melanoma patients treated with combined ipilimumab and anti-PD-1 immunotherapy.
